# Genome Sequences of Arthrobacter globiformis *B-2979* Phages GlobiWarming and TaylorSipht

**DOI:** 10.1128/mra.00923-22

**Published:** 2022-10-05

**Authors:** Miguel B. Bugayong, Aileen Cha, Carly L. Hamel, Ryan R. Johnson, James Kim, Joseph J. Kim, Andrew S. Levy, Kenneth D. Nguyen, Luc H. Pham, Anusha Sapre, Aidan C. Scanlan, Brandon Ye, Christa Bancroft

**Affiliations:** a Department of Biological Sciences, University of Southern California, Los Angeles, California, USA; b Department of Chemistry, University of Southern California, Los Angeles, California, USA; c Department of Health Promotion and Disease Prevention Studies, Keck School of Medicine of USC, Los Angeles, California, USA; d Department of Quantitative and Computational Biology, University of Southern California, Los Angeles, California, USA; Queens College CUNY

## Abstract

Phages GlobiWarming and TaylorSipht are siphoviruses isolated on Arthrobacter globiformis *B-2979*. GlobiWarming has a 42,691 bp long genome that encodes 74 genes, whereas TaylorSipht has a 39,051 bp genome that encodes 65 genes. Both phages encode functions typical of temperate phages, with each including an immunity repressor, integrase, and excise.

## ANNOUNCEMENT

Thought to be the most abundant biological entity on Earth, bacteriophages, or phages, are viruses that infect and replicate within bacterial hosts (1). Phages that infect *Arthrobacter*, a genus of primarily soil-dwelling bacteria that play important roles in bioremediation and in the promotion of plant growth, have been relatively unexplored (2). Further research regarding *Arthrobacter* phages could serve to uncover their ecological role in soil microenvironments.

**FIG 1:**
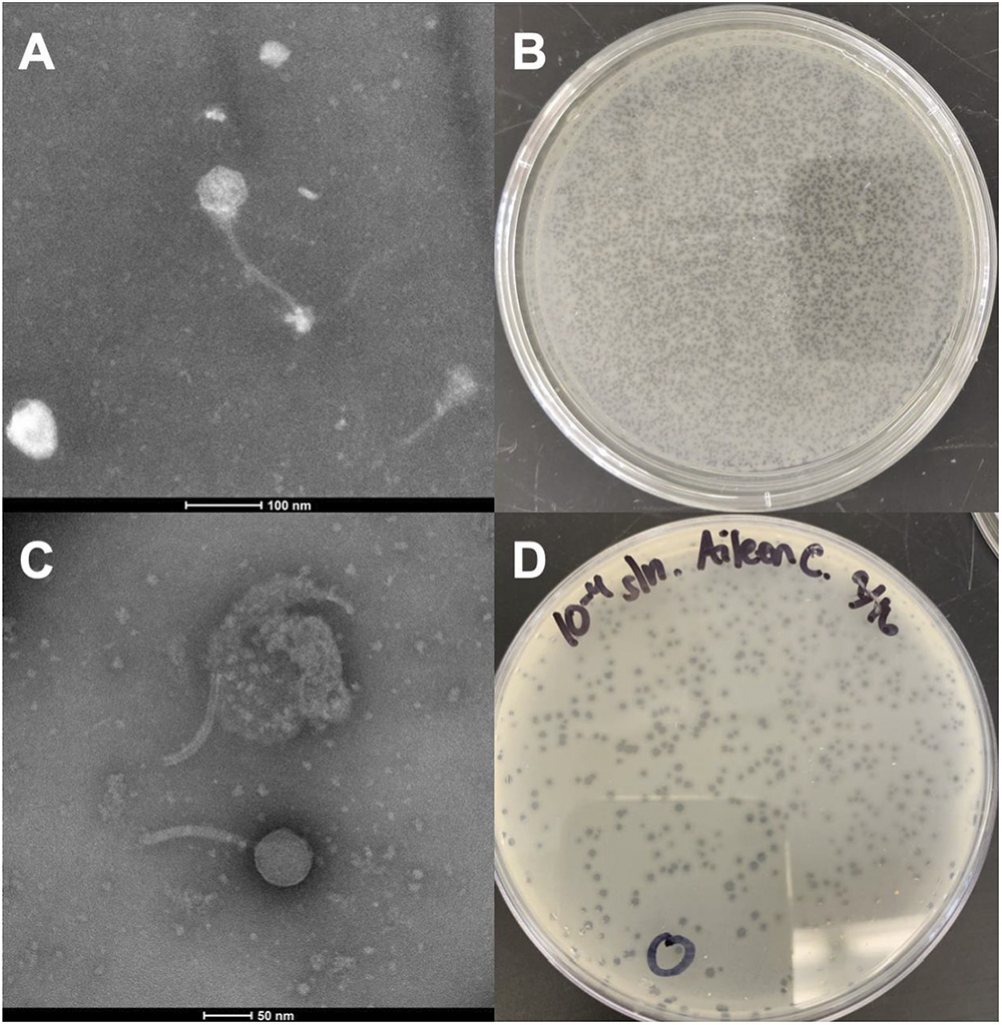
Transmission electron microscopy images and plaque assays for GlobiWarming (A and B) and TaylorSipht (C and D).

Phages GlobiWarming and TaylorSipht were isolated from damp soil samples one inch below the ground surface at the University of Southern California (USC) in Los Angeles, CA (34.021115 N, 118.287386 W and 34.02402 N, 118.2845 W, respectively) using standard procedures (https://seaphagesphagediscoveryguide.helpdocsonline.com/home). Briefly, each soil sample was washed in peptone-yeast extract-calcium (PYCa) medium. The wash was filtered (0.22 μm) and then inoculated with Arthrobacter globiformis *B-2979* and incubated with shaking at 30°C for 48 h. The culture was refiltered, diluted, and plated in soft agar containing Arthrobacter globiformis *B-2979*. After 24 h at 30°C, both GlobiWarming and TaylorSipht produced small clear plaques of approximately 1 mm in diameter (Fig. 1). Both phages were purified with 2 to 3 rounds of plating before being imaged via negative-stain transmission electron microscopy. Both phages have *Siphoviridae* structures, with GlobiWarming possessing a 207.3 to 214.4 nm tail and a capsid diameter of 59.2 to 60.4 nm and TaylorSipht possessing a 116.5 to 118.7 nm tail and a capsid diameter of 57.1 to 59.4 nm (*n* = 3) (Fig. 1).

Double-stranded DNA was purified using a Promega Wizard DNA Cleanup Kit, prepared for sequencing using a NEB Ultra II Kit, and sequenced on an Illumina MiSeq (v3 reagents). The sequencing reads, detailed in Table 1, were assembled using Newbler v2.9 and checked for accuracy and genomic termini using Consed v29. This yielded a 42,691 bp genome for GlobiWarming and a 39,051 bp genome for TaylorSipht, with both having 3′ single-stranded overhangs.

**TABLE 1:** Sequencing data and genome characteristics for GlobiWarming and TaylorSipht

Characteristic	GlobiWarming	TaylorSipht
No. of sequencing reads	581,462	910,779
Length of sequencing reads	150-base single-end	150-base single-end
Coverage of sequencing reads	2,000×	3,498×
Genome length	42,691 bp	39,051 bp
Guanine-cytosine %	65.0%	68.4%
Genome end types	3′ single-stranded overhangs (5′-CGCCGGAGA-3′)	3′ single-stranded overhangs (5′-GAGTCGCCGGCA-3′)
Cluster assignment	FA	AS

The genome sequences were auto-annotated using DNAMaster v5.23.6 (cobamide2.bio.pitt.edu) embedded with GeneMark v2.0 (3) and Glimmer v3.02 (4). Following auto-annotation, Starterator [http://phages.wustl.edu/starterator/] was used to refine the suggested start sites. GlobiWarming and TaylorSipht encode 74 and 65 open reading frames, respectively, and no tRNAs were identified by Aragorn v1.2.38 (5) or tRNAscanSE (6). The default parameters were used for all software tools employed. Based on a gene-content similarity of at least 35% to phages in the actinobacteriophage database (7), GlobiWarming and TaylorSipht are assigned to phage clusters FA and AS1. HHPred (8), NCBI BLASTp (9), and the Phamerator database Actino_Draft (10) were used to deduce the functions of open reading frames. Both phages encode an immunity repressor gene, a tyrosine integrase (GlobiWarming encodes two tyrosine integrases), and an excise. Therefore, both are likely to be temperate phages, despite them both forming clear plaques similar to those produced by other cluster FA and AS1 phages. The genomes of both phages are similarly organized, with structure and assembly genes occupying the left arm of each genome and DNA metabolism genes occupying the right arm. The majority of the genes are transcribed rightward, with the exception of a few genes in the center of each genome, including the immunity repressor, that are transcribed leftward. We hope to further explore how these phages are related to those within and outside their clusters by using phylogenetic tools.

### Data availability.

GlobiWarming is available at GenBank with accession no. ON970561 and at Sequence Read Archive (SRA) no. SRR18306084. TaylorSipht is available at GenBank with accession no. ON970604 and Sequence Read Archive (SRA) no. SRR18349669.
